# The importance of blood count and oxidative stress in the drug-naïve first episode schizophrenia

**DOI:** 10.1192/j.eurpsy.2022.2050

**Published:** 2022-09-01

**Authors:** J. Rog, M. Dzikowski, D. Juchnowicz, N. Waszkiewicz, A. Zalewska, M. Maciejczyk, H. Karakula-Juchnowicz

**Affiliations:** 1 Medical University of Lublin, 1st Department Of Psychiatry, Psychotherapy And Early Intervention, Lublin, Poland; 2 Medical University of Lublin, Department Of Psychiatric Nursing, Lublin, Poland; 3 Medical University of Bialystok, Department Of Psychiatry, Białystok, Poland; 4 Medical University of Bialystok, Experimental Dentistry Laboratory And Department Of Restorative Dentistry, Bialystok, Poland; 5 Medical University of Bialystok, Department Of Hygiene, Epidemiology And Ergonomics, Białystok, Poland

**Keywords:** Oxidative stress, First Episode Psychosis, white blood cells, drug-naïve

## Abstract

**Introduction:**

Schizophrenia (SZ) is associated with changes in haematological parameters related to low-grade inflammation state and could be amplified via oxidative stress (OS) related mechanisms. Although studies confirm this relationship, the results could be cofounded by patients’ treatment.

**Objectives:**

The study aimed to assess the connection between venous blood count and OS in drug-naïve first-episode SZ patients.

**Methods:**

The study consisted of 24 SZ drug-naïve patients during first episode of psychosis (median age: 22 years), and 31 healthy individuals (HC) as a control group (median age: 28 years). The examination included clinical data, OS parameters (enzymatic and non-enzymatic antioxidants), peripheral blood counts.

**Results:**

We did not find differences between SZ and HC in blood count parameters (p>0.05). In patients group, white blood cells (WBC), neutrophils and neutrophils-to-lymphocyte ratio (NLR) were positively related with the severity of positive symptoms (R=0.59, R=0.53, R=0.50; p<0.05, respectively). WBC was related to superoxide dismutase (SOD-1) levels (HC: R=-0.36, SZ: R=0.70; p<0.05). Neutrophils were positively related to catalase (CAT) (R=0.52; p<0.05) and ferric reducing antioxidant power (FRAP) (R=0.61; p<0.05), but only in the patients’ group. There was a positive relationship between NLR and CAT (R=0.45; p<0.05) in the SZ group.

**Conclusions:**

The results indicate potential connection and interplay between OS and blood count parameters in the onset of psychotic episode. Further studies on a larger group of patients are needed.

**Disclosure:**

No significant relationships.

